# Continuity of care and mortality for patients with chronic disease: an observational study using Norwegian registry data

**DOI:** 10.1093/fampra/cmad025

**Published:** 2023-04-19

**Authors:** Sahar Pahlavanyali, Øystein Hetlevik, Valborg Baste, Jesper Blinkenberg, Steinar Hunskaar

**Affiliations:** Department of Global Public Health and Primary Care, University of Bergen, Bergen, Norway; Department of Global Public Health and Primary Care, University of Bergen, Bergen, Norway; National Centre for Emergency Primary Health Care, NORCE Norwegian Research Centre, Bergen, Norway; Department of Global Public Health and Primary Care, University of Bergen, Bergen, Norway; National Centre for Emergency Primary Health Care, NORCE Norwegian Research Centre, Bergen, Norway; Department of Global Public Health and Primary Care, University of Bergen, Bergen, Norway; National Centre for Emergency Primary Health Care, NORCE Norwegian Research Centre, Bergen, Norway

**Keywords:** continuity of care, chronic disease, general practice, healthcare system, mortality, observational study

## Abstract

**Background:**

Research on continuity of care (CoC) is mainly conducted in primary care and has received little acknowledgment in other levels of care. This study sought to investigate CoC across care levels for patients with selected chronic diseases, along with its association with mortality.

**Methods:**

In a registry-based cohort study, patients with ≥1 consultation in primary or specialist healthcare or hospital admission with asthma, chronic obstructive pulmonary disease (COPD), diabetes mellitus, or heart failure in 2012 were linked to disease-related consultation data in 2013–2016. CoC was measured by Usual Provider of Care index (UPC) and Bice–Boxermann continuity of care score (COCI). Values equal to one were categorized into one group and the rest into three equal groups (tertiles). The association with mortality was determined by Cox regression models.

**Results:**

The highest mean UPC_total_ was measured for patients with diabetes mellitus (0.58) and the lowest for those with asthma (0.46). The population with heart failure had the highest death rate (26.5). In adjusted Cox regression analyses for COPD, mortality was 2.6 times higher (95% CI 2.25–3.04) for patients in the lowest tertile of continuity compared to those with UPC_total_ = 1. Patients with diabetes mellitus and heart failure showed similar results.

**Conclusion:**

CoC was moderate to high for disease-related contacts across care levels. A higher mortality associated with lower CoC was observed for patients with COPD, diabetes mellitus, and heart failure. A similar, but not statistically significant trend was found for patients with asthma. This study suggests that higher CoC across levels of care can decrease mortality.

**Key messages**

- Chronic care management in Norway is primarily carried out by GPs.
- Continuity of care for chronic disease is moderate to high across care levels.
- Lower continuity of care is associated with increased mortality.

## Background

There is an increasing demand for healthcare services worldwide, on account of aging populations and growing prevalence of chronic diseases.^1–3^ In Norway with 5.4 million inhabitants, 1.9 million patients received at least one episode of treatment at hospitals in 2020^4^ and on average each individual had 2.8 consultations with a general practitioner (GP).^5^ Older patients and those with chronic diseases comprise many of these contacts^6^ and may receive their healthcare needs from multiple providers at different levels.

CoC is defined as consistent care for individual patients over time.^7^ There are three main types of CoC: informational (availability of patient health records to providers), management (consistent care coordination between several providers), and personal (a therapeutic relationship between one patient and one or more providers).^7,8^ Personal continuity is considered as an essential pillar of general practice^8^ and is particularly beneficial for older people^9,10^ or patients with chronic or complex conditions.^1,11^ Major benefits of CoC for patients are increased usage of preventive care,^12^ reduction of adverse health outcomes,^11,13^ decreased risk of emergency visits and hospital admissions.^9,14–18^ CoC is also associated with reduced morbidity and mortality within sectors of healthcare systems like midwifery^19^ and primary care,^17,20–23^ but the impact across other sectors of healthcare and an overall healthcare system is still scarcely researched.^18,24,25^ This study aims to investigate personal continuity for patients with asthma, chronic obstructive pulmonary disease (COPD), diabetes, and heart failure in the overall healthcare system and whether continuity across the Norwegian healthcare system is associated with lower mortality.

## Methods

### Norwegian healthcare system

The Norwegian healthcare system with universal coverage is divided into primary and specialist services. The primary healthcare system including regular general practitioners (RGP), and out-of-hours (OHH) services is managed by municipalities, while four regional health trusts organize specialist healthcare, including hospitals (inpatient and outpatient care) and private specialists with public contracts (PSPC) which operate like hospital outpatient clinics.^26^ In 2001, the government introduced the RGP scheme giving residents the right to choose their RGP^27^ and by the start of 2021, 99% of Norwegian residents were assigned to an RGP list.^28^ RGPs and OOH services function as gatekeepers for specialist healthcare, and all access to specialist healthcare is referral-based.

### Study design

A registry-based cohort study was conducted by using data from several national healthcare and population registries, from the years 2012–2018.

### Data sources

We linked data from the following four registries by using a pseudo-id provided by Statistics Norway (SSB) for each patient’s national identification number:

- Control and Payment of Reimbursement to Health Service Providers database (KUHR)
- Norwegian Patient Registry (NPR)
- Norwegian RGP registry, and
- Statistics Norway (SSB)

KUHR contains claims data from RGPs and OOH services. For this study, we used data regarding consultations and home visits. Information about each patient’s registered RGP was obtained from the RGP registry. NPR contains all patients’ contacts with specialist healthcare. We included both inpatients and patients with day cases for hospital admissions, but only those with somatic conditions.

Physicians in Norwegian primary care use the International Classification of Primary Care, second edition (ICPC-2) to code diagnoses, while NPR contains codes from The International Statistical Classification of Diseases and Related Health Problems version 10 (ICD-10).

Data on patients’ date of birth, sex, date of death, centrality classes (urban/rural),^29^ and educational levels^30^ were obtained from SSB.

### Study population

We identified all patients with ≥1 consultation with GP, OOH services, PSPCs, hospital outpatient clinics, or hospital admission with a diagnosis code for asthma, chronic obstructive pulmonary disease (COPD), diabetes mellitus, or heart failure, during 2012. One patient sample for each diagnosis was defined irrespective of the others, and a patient could belong to several populations (there was a maximum of 2% overlap between any two or more populations). These patients were linked to data from KUHR, NPR, and RGP registries during 2013–2016, and consultations with these diagnoses from general practices, OOH services, PSPCs, and hospital outpatient clinics were labelled.

We used four years (2013–2016) to measure CoC prior to the period observing mortality, as we wanted to study continuity using a long-time perspective. Additionally, this was done to reduce a possible impact of changes in contact patterns in the last period of life for those who died. Therefore, those who died in the period estimating CoC are not included in the study populations. Patients with less than four consultations with asthma, COPD, diabetes mellitus, or heart failure, during 2013–2016 were also excluded. We also required ≥1 all-cause consultation with RGP practice in 2016 to exclude those who had moved to a nursing home during the last observation year, since we did not have data on health care use for those in nursing homes.

A provider mainly refers to one physician, but in few cases, it is assigned to an organization. Since claims data from PSPCs, RGPs, and the majority of OOH services contain physicians’ identification number we could identify each RGP, GP, PSPC, and most physicians at OOH services as individual providers. However, claims from hospital outpatient clinics, and a few OOH services are registered with organizations’ name and ID, and each of these organizations was regarded as one provider.

### Continuity of care

We measured CoC based on the number of consultations each patient experienced with different physicians for a specific disease. We used *The Usual Provider of Care index (UPC)* and *Bice–Boxermann continuity of care score (COCI)*^31^ as they are preferred measures for claims-based data.^9^ We calculated UPC as a proportion of RGP consultations in the overall healthcare system (*UPC*_*total*_):


$$\begin{array}{l} UPC_{total}=\frac{RGP\;consultations\;(n)}{total\;number\;of\;consultations\;(N_{total})}. \end{array}$$

COCI, exhibiting the dispersion and distribution of all visits to all providers,^32^ was measured in the overall healthcare system including all consultations with all providers:


$$\begin{array}{l} COCI_{total}=\frac{\overset{\;p}{\underset{i=1}{\sum }}n_{i}^{2}-N}{N(N-1)}, \end{array}$$

where *N* is the total number of visits, *n*_*i*_ is the total number of visits to *i*th provider, and p is the total number of providers.

Both indices are measured on a scale from 1 to 0.^32,33^ The index value of 1.0 represents maximum continuity and was categorized as one group, and the index values <1.0 were divided into three groups with an equal number of patients (tertiles). The proportion of patients in UPC_total_ tertile groups for COPD and heart failure was evenly distributed, but in asthma and diabetes mellitus many patients in some of the tertiles had the same value for UPC causing an uneven distribution of patients in tertile groups. We also defined a variable by combining the categories with maximum and high tertile from COCI, and moderate and low tertile from UPC.

### Mortality

Mortality was investigated in 24 months for deaths during 2017 and 2018. We included all-cause mortality as we did not have access to the specific causes of death.

### Study covariates

Age, sex, centrality index, educational level, number of consultations with each provider, number of providers for each patient during 2013–2016, and the ICPC morbidity index^34^ (0, 1, 2, and 3 or more) were used as covariates.

Statistics Norway categorizes municipalities into six levels (centrality index), level one containing the most central (urban) areas, and level six the least central (rural) areas.^29^ SSB also classifies educational level into three main groups, low (elementary school or less), medium (upper secondary school), and high (university and higher education) based on the highest fulfilled education.^30^

### Statistical analyses

Frequency and percentages were carried out for the covariates for each population. The number of providers and disease-related consultations with each provider were also calculated. Distribution of indices was displayed by mean and boxplot (median, interquartile range, minimum, and maximum values).

A Cox regression model was performed for each population separately to investigate the association between CoC and mortality. Patients were followed for 24 months or until death. Time to death was calculated in days. *UPC*_*total*_*and COCI*_*total*_ were independent variables. Both crude and adjusted analyses were performed. In the adjusted analyses, we included the patient’s age (continuous), sex, centrality index, the total number of disease-related consultations in 2013–2016, and comorbidity index. We did not include the educational level variable in the adjusted model since this variable had many missing values for asthma (24%) and the estimates changed marginally in the adjusted analyses for this variable in other populations. Other covariates had very few missing (<0.01%). Hazard ratio and 95% confidence interval (CI) were calculated. All analyses were conducted using Stata version 16.1.

## Results

**Fig. 1.**
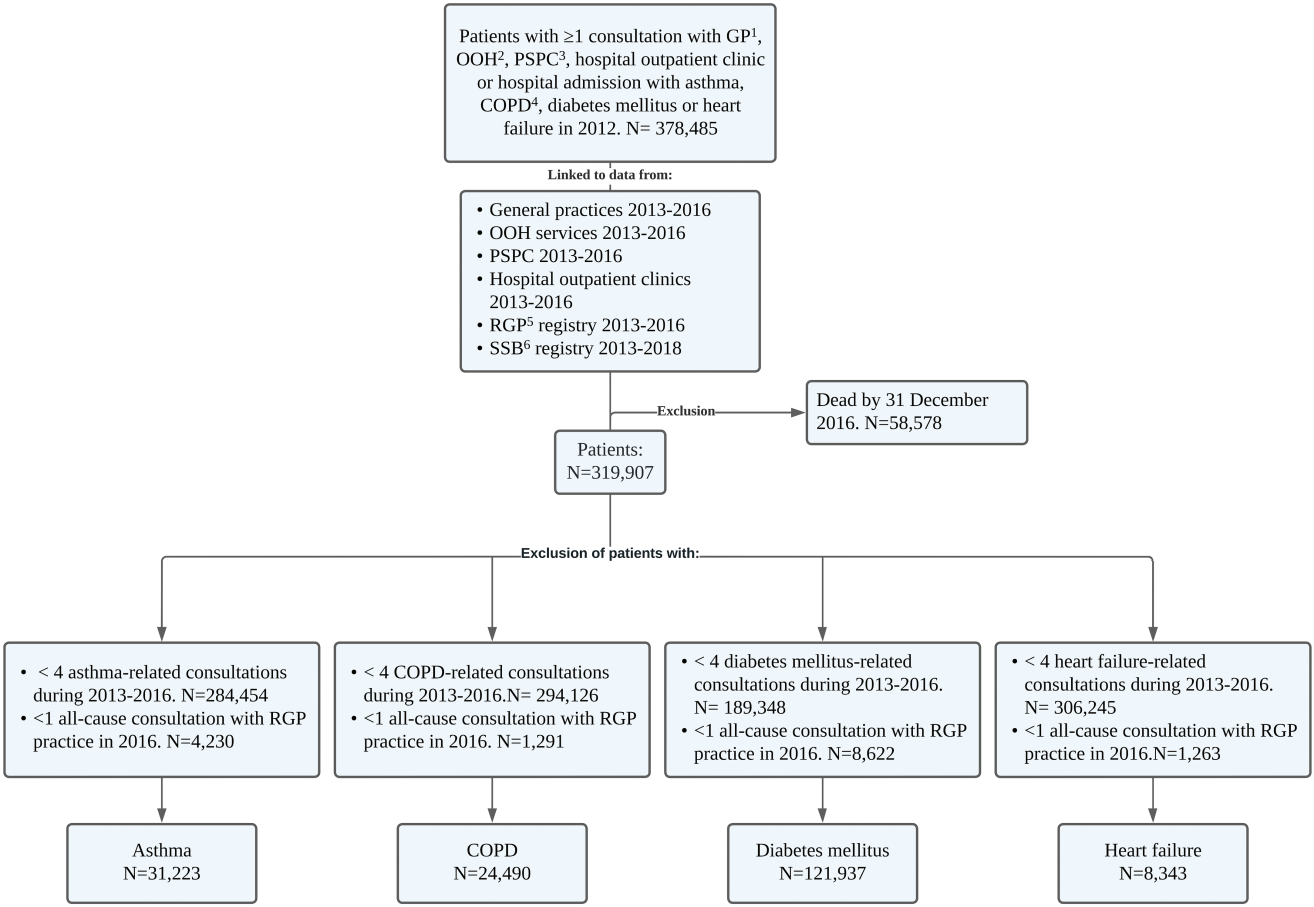
Flow chart presenting the inclusion and exclusion process and number of patients in each study population. Patients were identified in 2012 and met the criteria for ≥4 disease-related consultations in 2013–2016 for the 4 chosen diseases: asthma, chronic obstructive pulmonary disease, diabetes mellitus, and heart failure and ≥1 all-cause consultations with GP practice in 2016. Patients with more than one of the chronic conditions were included in all relevant study populations. ^1^GP: general practitioner ^2^OOH: out-of-hour service ^3^PSPC: private specialist with public contracts ^4^COPD: chronic obstructive pulmonary disease ^5^RGP: regular general practitioner ^6^SSB: Statistics Norway.

We identified 378,485 patients with at least one consultation in primary or specialist healthcare or at least one hospital admission in 2012. After applying the inclusion and exclusion criteria, the four populations were defined: asthma (*N* = 31,223), COPD (*N* = 24,490), diabetes mellitus (*N* = 121,937), and heart failure (*N* = 8,343) (Fig. 1).

Table 1 displays descriptive features of the four study populations. Patients with diabetes mellitus constituted the largest population. Those with heart failure formed the smallest and oldest study population with a mean age of 79 years and 90% of patients ≥65 years old. Fifty-eight percent of patients with asthma were female in contrast to diabetes mellitus (45%), and heart failure (39 %).

**Table 1.** Patients’ characteristics in the four study populations: asthma, chronic obstructive pulmonary disease, diabetes mellitus and heart failure, 2013–2016.

Diagnoses	Asthma		COPD^a^		Diabetes mellitus		Heart failure	
Characteristics	Patients	Consultations	Patients	Consultations	Patients	Consultations	Patients	Consultations
Total (*N*)	31,223	274,584	24,490	374,948	121,937	2,023,030	8,343	127,844
Mean age (years)	45		73		68		79	
≥65 years old (%)	28.8	29.8	82.8	83.7	61.6	59.3	87.5	86.7
Female (%)	57.5	59.7	50.5	52.0	44.5	44.5	39.3	36.4
Centrality index^b^ (%)								
1 (most urban)	20.8	20.9	14.4	14.7	16.5	17.6	16.2	17.0
2	24.6	24.8	24.5	25.5	23.2	23.5	22.7	23.7
3	26.2	26.4	27.0	27.3	26.2	26.7	28.3	28.6
4	18.1	17.7	20.3	19.7	19.7	19.6	18.8	18.4
5	7.6	7.6	9.5	8.9	10.0	9.2	9.8	8.6
6 (most rural)	2.7	2.5	4.2	3.9	4.0	3.4	4.2	3.6
Educational level^c^ (%)								
Low	26.2	26.5	44.2	45.1	34.0	34.7	37.8	37.0
Medium	31.2	32.3	46.2	45.8	46.3	46.0	46.5	47.4
High	18.7	19.4	8.8	8.2	18.1	17.6	14.7	14.6
Missing	23.9	21.7	0.8	0.9	1.6	1.8	1.0	1.0
Comorbidity groups^d^ (%)								
0	72.6		2.6		2.4		8.4	
1	20.2		55.2		71.0		44.2	
2	5.7		30.2		21.0		33.1	
≥3	1.5		12.0		5.7		14.3	
Proportion of patients who visited								
RGP	77.2		89.9		92.8		81.3	
Other GPs	38.2		49.6		39.1		37.6	
OOH	18.1		29.3		5.4		12.0	
Hospital outpatient	43.6		72.0		60.6		74.2	
PSPC	34.3		17.8		54.4		7.5	
Proportion of patients with visits limited to								
One provider	28.8		15.9		12.6		26.6	
RGP	15.7		11.8		10.3		14.2	
Other GPs	0.8		0.6		0.4		0.7	
OOH	0.0		0.0		0		0	
Hospital outpatient	4.0		2.6		1.2		11.1	
PSPC	8.3		0.9		0.7		0.6	
Two providers	39.9		32.8		38.1		40.7	
Three or more providers	31.3		51.3		49.3		32.7	

^a^COPD, chronic obstructive pulmonary disease.

^b^Centrality index 1 represents the most urban and 6 the most rural. This variable has 0.1% missing for asthma and diabetes.

^c^Educational level: low (elementary school or less), medium (upper secondary school), and high (university and higher education).

^d^Number of comorbidity groups based on ICPC morbidity index.^34^

### Utilization of healthcare

In all four populations, RGPs were the most consulted providers (asthma: 77.2%, COPD 89.9%, diabetes mellitus 92.8%, and heart failure 81.3%) followed by hospital outpatient clinics (Table 1). The asthma population had the highest number of patients who only visited one provider (29%), followed by heart failure (27%), COPD (16%), and diabetes mellitus (13%). About 50% of patients with COPD and diabetes mellitus consulted three or more providers, in contrast to less than 30% of patients with asthma and heart failure.

**Fig. 2.**
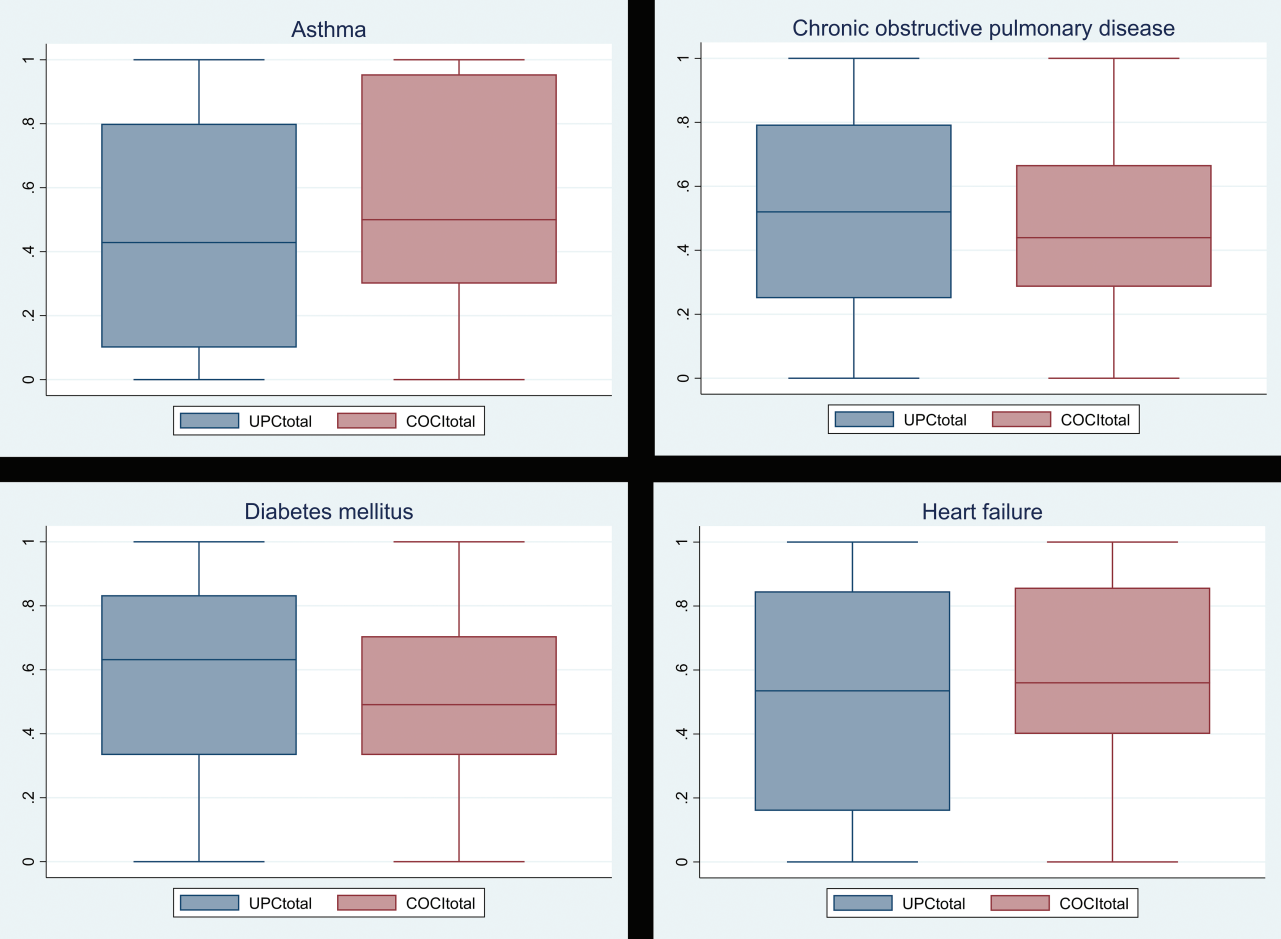
Box plot showing distribution of continuity indices. Total UPC (UPC_total_) and total COCI (COCI_total_) measured for patients with asthma, chronic obstructive pulmonary disease, diabetes mellitus, and heart failure. Each box represents median, interquartile range, minimum, and maximum values.

The UPC_total_ measured for these patients were as follows: asthma (mean: 0.46, SD: 0.36), COPD (mean: 0.52, SD: 0.32), diabetes mellitus (mean: 0.58, SD: 0.31), and heart failure (mean: 0.51, SD: 0.36) (Fig. 2).

The COCI_total_ values were found to be: asthma (mean: 0.56, SD: 0.31), COPD (mean: 0.49, SD: 0.27), diabetes mellitus (mean: 0.53, SD: 0.25), and heart failure (mean: 0.61, SD: 0.27) (Fig. 2).

High COCI and low UPC indicating continuity with another provider than the RGP was measured for 21% (33% among patients ≤ 18) of patients with asthma, and 17% (72% ≤ 18) of those with heart failure, 11% (64% ≤ 18) of the population with COPD and 7% (71% ≤ 18) of patients with diabetes mellitus.

### Continuity of care and mortality

The population with heart failure had the highest proportion of deaths (26.5%), followed by COPD (16.7%), diabetes mellitus (5.9%), and asthma (2.1%) (Table 2).

**Table 2.** Mortality by continuity of care measured in the overall healthcare system by UPC_total_^a^ for patients with asthma, chronic obstructive pulmonary disease, diabetes mellitus, and heart failure (Norway, 2017–2018).

	Patients		Death		Mortality			
					Crude		Adjusted^b^	
Diagnoses	*N*	(%)	*N*	(%)	HR	95% CI	HR	95% CI
Asthma	31,223	(100)	651	(2.1)				
Maximum (UPC = 1)	4,888	(15.7)	139	(2.8)	ref.		ref.	
High (third tertile, 0.50 < UPC ≤ 0.99)	8,279	(26.5)	209	(2.5)	0.89	(0.72–1.09)	0.95	(0.77–1.18)
Moderate (second tertile, 0.14 < UPC ≤ 0.50)	9,492	(30.4)	172	(1.8)	**0.63**	**(0.51**–**0.79)**	0.99	(0.79–1.24)
Low (first tertile, UPC ≤ 0.14)	8,564	(27.4)	131	(1.5)	**0.53**	**(0.42**–**0.68)**	0.99	(0.78–1.26)
COPD^c^	24,490	(100)	4,081	(16.7)				
Maximum (UPC = 1)	2,890	(11.8)	202	(6.7)	ref.		ref.	
High (third tertile, 0.62 < UPC ≤ 0.99)	7,255	(29.6)	964	(13.3)	**1.96**	**(1.69**–**2.29)**	**1.62**	**(1.39**–**1.89)**
Moderate (second tertile, 0.31<UPC ≤0.62)	7,149	(29.2)	1,394	(19.5)	**2.98**	**(2.57**–**2.45)**	**2.41**	**(2.06**–**2.77)**
Low (first tertile, UPC≤0.31)	7,196	(29.4)	1,521	(21.1)	**3.26**	**(2.81**–**3.78)**	**2.63**	**(2.25**–**3.04)**
Diabetes mellitus	121,937	(100)	7,219	(5.9)				
Maximum (UPC = 1)	12,515	(10.3)	603	(4.8)	ref.		ref.	
High (third tertile, 0.71 < UPC ≤ 0.99)	37,771	(31.0)	1,989	(5.3)	**1.09**	**(1.0**–**1.20)**	1.06	(0.96–1.16)
Moderate (second tertile, 0.40 < UPC ≤ 0.71)	35,187	(28.9)	2,221	(6.3)	**1.32**	**(1.21**–**1.44)**	**1.23**	**(1.12**–**1.35)**
Low (first tertile, UPC ≤ 0.40)	36,464	(29.9)	2,406	(6.6)	**1.38**	**(1.26**–**1.51)**	**1.59**	**(1.44**–**1.74)**
Heart failure	8,343	(100)	2,207	(26.5)				
Maximum (UPC = 1)	1,188	(14.2)	259	(21.8)	ref.		ref.	
High (third tertile, 0.64 < UPC ≤ 0.99)	2,408	(28.9)	702	(29.2)	**1.38**	**(1.20**–**1.59)**	**1.30**	**(1.12**–**1.50)**
Moderate (second tertile, 0.21 < UPC ≤ 0.64)	2,368	(28.4)	695	(29.4)	**1.39**	**(1.21**–**1.61)**	**1.66**	**(1.43**–**1.92)**
Low (first tertile, UPC ≤ 0.21)	2,379	(28.5)	551	(23.2)	1.06	(0.91–1.22)	**1.64**	**(1.41**–**1.92)**

UPC_total_ is divided into four groups. UPC=1 represents the maximum value in the first group. The index values <1 are divided into tertiles (high, moderate and low). Values in bold text present *P < 0.05.*

^a^UPC_total_ = usual provider of care index in overall healthcare system.

^b^Adjusted for sex, age (continuous), centrality index, total number of disease-related consultations in four years, and comorbidity groups (based on ICPC morbidity index)^34^ in a Cox regression model.

^c^COPD, chronic obstructive pulmonary disease.

Being in the lowest tertile of continuity in patients with COPD, diabetes mellitus, or heart failure was associated with higher mortality. For instance, mortality in the lower tertile (UPC ≤ 0.31) for patients with COPD was 2.6 times higher (95% CI 2.25–3.04) than for patients with UPC_total_ = 1, in the adjusted model. Sensitivity analyses for UPC_total_ and mortality restricted to patients ≥65 years showed the same patterns for all populations (results not shown).

When examining the association between COCI_total_ and mortality (Table 3), the risk of death increased as COCI was reduced for patients with COPD, diabetes mellitus, and heart failure.

**Table 3.** Mortality by continuity of care measured in the overall healthcare system by COCI_total_^a^ for patients with asthma, chronic obstructive pulmonary disease, diabetes mellitus, and heart failure (Norway, 2017–2018).

	Patients		Death		Mortality			
					Crude		Adjusted^b^	
Diagnoses	*N*	(%)	*N*	(%)	HR	95% CI	HR	95% CI
Asthma	31,223	(100)	651	(2.1)				
Maximum (COCI = 1)	7,788	(24.9)	184	(2.4)	ref.		ref.	
High (third tertile)	6,998	(22.4)	140	(2.0)	0.85	(0.68–1.05)	0.81	(0.66–1.03)
Moderate (second tertile)	9,255	(29.6)	206	(2.3)	0.94	(0.77–1.15)	1.07	(0.87–1.30)
Low (first tertile)	7,182	(23.0)	121	(1.7)	0.71	(0.56–0.89)	0.89	(0.70–1.12)
COPD^c^	24,490	(100)	4,081	(16.7)				
Maximum (COCI = 1)	3,354	(13.7)	281	(8.4)	ref.		ref.	
High (third tertile)	7,058	(28.8)	1,032	(14.6)	**1.80**	**(1.58**–**2.06)**	**1.42**	**(1.24**–**1.62)**
Moderate (second tertile)	7,040	(28.8)	1,428	(20.3)	**2.57**	**(2.27**–**2.92)**	**1.99**	**(1.75**–**2.27)**
Low (first tertile)	7,038	(28.7)	1,340	(19.0)	**2.41**	**(2.12**–**2.74)**	**1.92**	**(1.68**–**2.18)**
Diabetes mellitus	121,937	(100)	7,219	(5.9)				
Maximum (COCI = 1)	13,059	(10.7)	654	(5.0)	ref.		ref.	
High (third tertile)	36,116	(29.6)	1,868	(5.2)	1.03	(0.95–1.13)	1.04	(0.94–1.13)
Moderate (second tertile)	36,666	(30.1)	2,279	(6.2)	**1.25**	**(1.14**–**1.36)**	**1.21**	**(1.11**–**1.32)**
Low (first tertile)	36,096	(29.6)	2,418	(6.7)	**1.35**	**(1.24**–**1.47)**	**1.24**	**(1.13**–**1.35)**
Heart failure	8,343	(100)	2,207	(26.5)				
Maximum (COCI = 1)	1,791	(21.5)	355	(19.8)	ref.		ref.	
High (third tertile)	2,223	(26.7)	611	(27.5)	**1.44**	**(1.27**–**1.64)**	**1.23**	**(1.08**–**1.41)**
Moderate (second tertile)	2,256	(27.0)	622	(27.6)	**1.43**	**(1.26**–**1.63)**	**1.37**	**(1.20**–**1.56)**
Low (first tertile)	2,073	(24.9)	619	(29.9)	1.59	(1.39–1.81)	**1.51**	**(1.32**–**1.73)**

COCI_total_ is divided into four groups. COCI = 1 represents the maximum value in the first group. The index values <1 are divided into tertiles (high, moderate, and low). Values in bold text present *P < 0.05*.

^a^COCI_total_ = Bice–Boxerman continuity of care score.

^b^Adjusted for sex, age (continuous), centrality index, total number of disease-related consultations in four years, and comorbidity groups (based on ICPC morbidity index)^34^ in a Cox regression model.

^c^COPD, chronic obstructive pulmonary disease.

## Discussion

In this study investigating CoC and mortality for patients with asthma, COPD, diabetes mellitus, and heart failure in Norway, we found that most of the disease-related consultations were with RGPs. Additionally, we observed higher mortality associated with lower CoC in the overall healthcare system, for patients with COPD, diabetes mellitus, and heart failure, but not for asthma.

### Utilization of healthcare

Our findings are consistent with earlier literature,^35,36^ confirming that RGPs in Norway have a major role in the care for patients with chronic diseases. Despite almost 80% of patients visiting their RGPs, around 50% of patients with COPD and diabetes mellitus and a third of asthma and heart failure populations had visited three or more providers. Hospital outpatient clinics were the second most visited, also for visits exclusive to one provider only. Since these consultations are specific for each diagnosis, we assume that, after RGPs, the specialist healthcare services have a substantial proportion of disease-related contacts, and that severity of illness is a plausible explanation for this.

### Continuity of care and mortality

Patients with these chronic diseases have moderate to high CoC for disease-related contacts, supporting the results from a previous study that measured CoC during a shorter period.^31^ Our results showing lower mortality with higher CoC with RGP is also in line with findings from a recent Norwegian study.^23^ The two studies defined and investigated CoC and its association with mortality by different methods and in distinct levels of care, but they reached similar conclusions.

More frequent use of specialist care could be associated with more severe disease, which itself may result in higher mortality, thus overestimating the effect of CoC on mortality, especially as measured by UPC. To reduce this possible bias, we adjusted for total number of disease-related consultations as a proxy for severity.

Comorbidity might also increase mortality, therefore we adjusted for the number of comorbidities to reduce this potential bias, by using the ICPC morbidity index.^34^

Patients with high COCI and low UPC, having higher CoC with specialist healthcare, mainly belong to the following two categories: the largest category comprised of patients ≤18 years and without any comorbidities who more often had diagnosis-related follow-up with specialist healthcare and not by GPs. In Norway, it is an established practice that follow-ups for children with these chronic diseases are carried out by paediatricians. The other category comprised older patients with higher continuity with specialist healthcare probably due to the severity of their disease.

Increased mortality associated with a decreased level of CoC was found for patients with COPD, diabetes mellitus, and heart failure, in accordance with earlier findings.^20,23^ However, in the case of asthma, there was no significant association between continuity and mortality in adjusted analyses, and the unadjusted analyses for UPC_total_ showed an inverse relationship with mortality. We believe that this finding is due to a young (mean age = 45) and healthy (73% with no comorbidities) population with low total death rate (2.1%), and that the observed effect in the unadjusted analyses was caused by age as a confounding factor.

While many studies may acknowledge the importance of CoC in primary care, there are few studies assessing CoC both in primary and specialist healthcare systems. One study examined and compared CoC in primary and specialist healthcare simultaneously with a focus on care fragmentation within each speciality rather than across specialties.^18^ Our study is different in a way that we have studied CoC within the overall healthcare system. Despite the two different approaches, findings from both studies encourage CoC with the same RGP or specialist physician as we believe continuity in primary and specialist healthcare would reduce care fragmentation and lead to better patient outcome.

### Strengths and limitations

The study is based on data obtained from national registries, with the whole Norwegian population and a long observation period, thus avoiding selection bias. We chose some of the most common chronic conditions^37^ managed by both RGPs and specialist healthcare, and we believe that our findings are representative for the majority of chronic diseases. However, we suspect that our study populations consist of the most severely ill patients as a result of our strict inclusion criteria. They comprise a smaller proportion of the source populations for each disease, but their high utilization of healthcare services makes them optimal study populations.

Another limitation is that all hospital outpatient clinics and one OOH service each were included as an individual provider, resulting in an overestimation of COCI for these providers. The reality is that patients may meet different physicians at these healthcare services. This would dilute the effect of CoC on mortality. One might also argue that we thus do not actually measure personal continuity in this study, but we believe this type of continuity suits this study best.

Lack of access to the cause of death and the fact that mortality could be due to causes unrelated to the diseases under investigation might be a potential limitation. Additionally, we did not have access to data on the severity of the disease apart from the number of disease-related consultations which we used in the adjusted analyses as a proxy for severity. This might be a limitation since we do not know whether high CoC is due to severity which might be associated with higher mortality, and if so the effect of CoC may be underestimated.

To the best of our knowledge, there are no Norwegian studies determining the utilization of levels of healthcare services by patients with chronic diseases or examining the association between CoC and mortality simultaneously in primary and specialist healthcare. Therefore, we believe this study adds to previous literature with new approaches and our results may be mostly transferable to countries with a GP scheme and where primary care functions as a gatekeeper for specialist healthcare.

## Conclusions

Our results show that the majority of patients with asthma, COPD, diabetes mellitus, and heart failure have moderate to high CoC with RGP and hospital outpatient clinics. We observed higher mortality associated with lower CoC across all levels of care for patients with COPD, diabetes mellitus, and heart failure. With the increasing prevalence of chronic diseases in most countries, challenges in providing consistent and coordinated care will also rise. We believe a better understanding of CoC, and its benefits would help policymakers to organize healthcare systems to promote CoC both within and across primary and specialist healthcare services. This would improve patient care, particularly for those with the greatest needs.

## Supplementary Material

cmad025_suppl_Supplementary_Material

## Data Availability

Data were provided by HELFO, NPR, and SSB. Due to restrictions by the Norwegian Data Protection Authority, we cannot share the data publicly.
